# Praiseworthy Action

**Published:** 1870-01

**Authors:** 


Praiseworthy Action.—Massachusetts has established a State Board of Health. Drs. Henry I. Bowditch and George Derby have been chosen chairman and secretary respectively, and from an address made by the former at the first meeting of the board we learn the specific duties imposed upon it. These are (we quote Dr. Bowditch): "To take cognizance of everything tending to public health," and, consequently, "to endeavor to eradicate everything tending to public disease and death;" secondly, "to diffuse among the people a knowledge of the means of obtaining individual and public health and preventing disease;" lastly, "to investigate the effects of the use of intoxicating liquors upon the industry, prosperity, happiness, health and lives of the people," and to suggest legislation on any or all of the subjects submitted for enquiry to the board. Dr. Bowditch proposes for the diffusion of knowledge to use the lyceum, "the publication in a compact form and the wide circulation of the pith, so to speak, of our general knowledge on public hygiene," and by brief, business-like, unrhetorical reports annually to the legislature.

We hail this movement with applause, especially that portion of it which looks to the distribution of sound medical knowledge among the people. The obscurantists of our profession have had their way long enough, and done harm enough. It is now time that some one besides itinerant quacks should circulate knowledge on physiology and hygiene.

